# Supplementary material for: South China Sea documents the transition from wide continental rift to continental break up
Source: Nat Commun. 2020 Sep 11;11:4583. doi: 10.1038/s41467-020-18448-y (PMC7486367; doi:10.1038/s41467-020-18448-y)
Supplement: Supplementary file 1 — Supplementary Information [file 41467_2020_18448_MOESM1_ESM.pdf]

Supplementary Information

South China Sea documents the transition from wide continental rift to continental break up

– **Hongdan et al.**

Supplementary Fig. 1 | Structure elements of the northern South China Sea passive margin showing the location of the Baiyun and Liwan supradetachment sub-basins.

Supplementary Fig. 2 | Reflection data of the X–X' seismic line.

Supplementary Fig. 3 | Seismic cross-section showing the dome structure and detachment fault in the southern flank of the dome.

Supplementary Data 1 Secondary fault orientation

Supplementary Data 2 Grooves orientation

Supplementary Data 3 MCC dimensions and aspect ratios plot

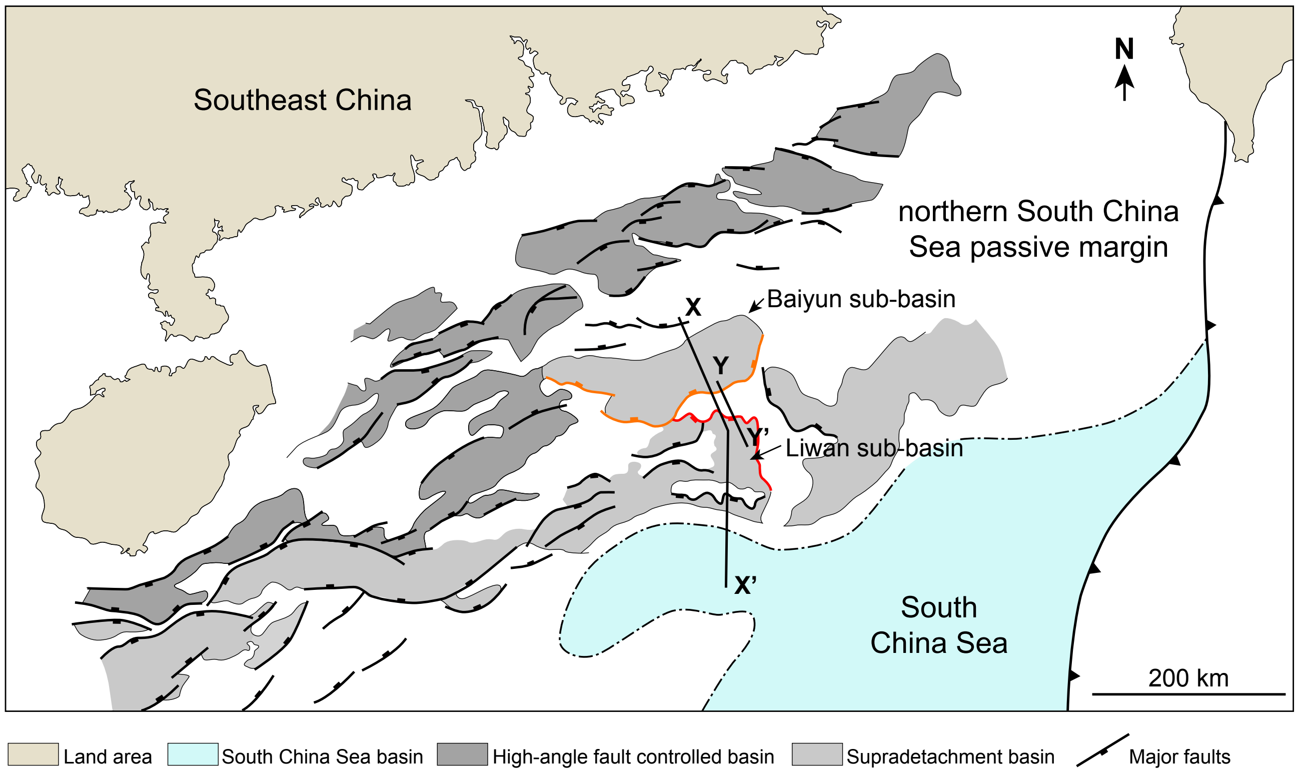

Supplementary Fig. 1 | Structure elements of the northern South China Sea passive margin showing the location of the Baiyun and Liwan supradetachment sub-basins. The Baiyun detachment fault and the Liwan detachment fault are denoted in orange and red, respectively. Note the divergent detachment fault are separated by a structural high that has no significant sediment deposition. X–X' is shown in Figure S2 and Y–Y' is shown in Supplementary Figure 3.

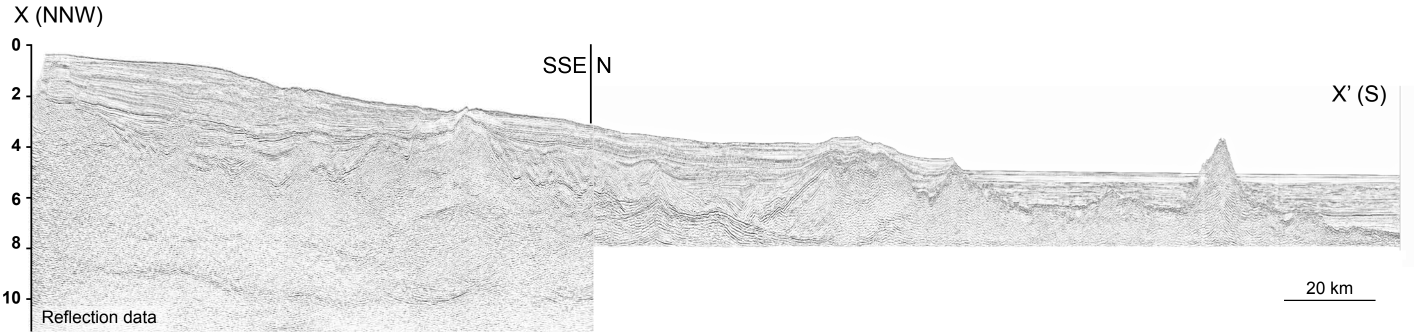

Supplementary Fig. 2 | Reflection data of the X–X' seismic line. Location of the seismic line is shown in Figure S1 and detailed interpretation is shown in Figure 1B.

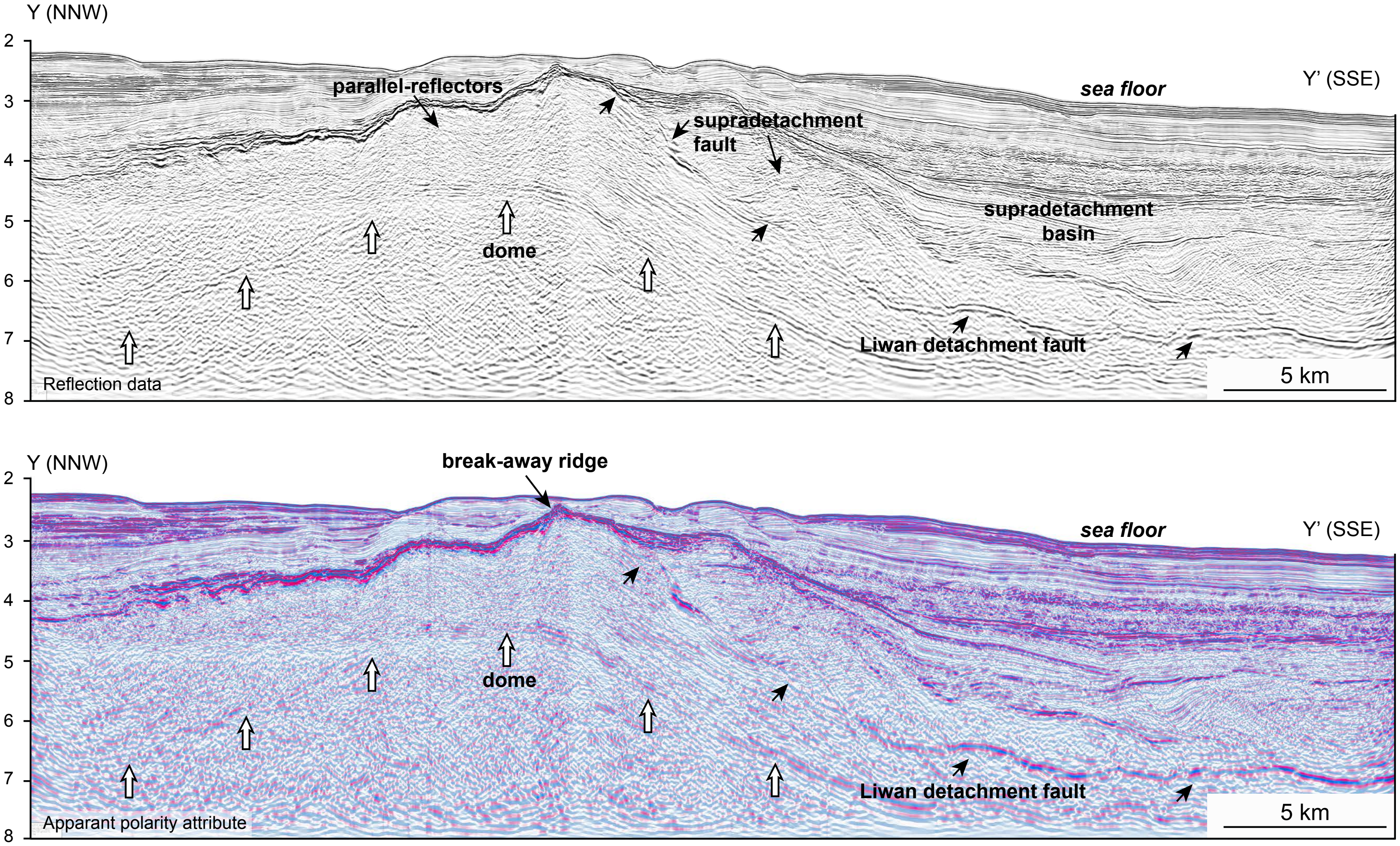

Supplementary Fig. 3 | Seismic cross-section showing the dome structure and detachment fault in the southern flank of the dome (see Supplementary Figure 1 for location). Note that the break-away ridge is the highest point in the footwall. Areas between the upper surface of the dome and the detachment show parallel-reflectors evocative of mylonite zone.
