# Supplementary material for: South China Sea documents the transition from wide continental rift to continental break up
Source: Nat Commun. 2020 Sep 11;11:4583. doi: 10.1038/s41467-020-18448-y (PMC7486367; doi:10.1038/s41467-020-18448-y)
Supplement: Supplementary file 3 — Description of Additional Supplementary Files [file 41467_2020_18448_MOESM3_ESM.pdf]

Description of Additional Supplementary Files

File Name: Supplementary Data 1
Description: Secondary fault orientation

File Name: Supplementary Data 2
Description: Grooves orientation

File Name: Supplementary Data 3
Description: MCC dimensions and aspect ratios plot
